# Sphingosine-1-Phosphate Prevents Egress of Hematopoietic Stem Cells From Liver to Reduce Fibrosis

**DOI:** 10.1053/j.gastro.2017.03.022

**Published:** 2017-07

**Authors:** Andrew King, Diarmaid D. Houlihan, Dean Kavanagh, Debashis Haldar, Nguyet Luu, Andrew Owen, Shankar Suresh, Nwe Ni Than, Gary Reynolds, Jasmine Penny, Henry Sumption, Prakash Ramachandran, Neil C. Henderson, Neena Kalia, Jon Frampton, David H. Adams, Philip N. Newsome

**Affiliations:** 1 Birmingham Liver Biomedical Research Unit, National Institute for Health Research, Centre for Liver Research, University of Birmingham, Birmingham, United Kingdom; 2 Centre for Cardiovascular Sciences, College of Medical and Dental Sciences, University of Birmingham, Birmingham, United Kingdom; 4 School Institute of Immunology and Immunotherapy, College of Medical and Dental Sciences, University of Birmingham, Birmingham, United Kingdom; 3 Medical Research Council Centre for Inflammation Research, University of Edinburgh, Edinburgh, United Kingdom

**Keywords:** Mouse Model, CCR2, Sphingolipid, Immune Cell Localization, α-SMA, α-smooth muscle actin, APC, allophycocyanin, BM, bone marrow, CCL, chemokine (C-C motif) ligand, CFSE, carboxyfluorescein succinimidyl ester, CLP, common lymphoid progenitors, CMP, common myeloid progenitors, DiR, 1,1′-dioctadecyl-3,3,3′,3′-tetramethylindotricarbocyanine iodide, HPC-7, hematopoietic progenitor cell line, HSC, hematopoietic stem cell, KSL, c-kit^+^ sca-1^+^ and lineage^neg^, MMP, matrix metalloproteinase, PSR, picrosirius red, SGPL1, sphingosine-1-phosphate lyase, SGPP1, sphingosine-1-phosphate phosphatase, S1P, sphingosine 1-phosphate, SphK1, sphingosine kinase 1

## Abstract

**Background & Aims:**

There is growing interest in the use of bone marrow cells to treat liver fibrosis, however, little is known about their antifibrotic efficacy or the identity of their effector cell(s). Sphingosine-1-phosphate (S1P) mediates egress of immune cells from the lymphoid organs into the lymphatic vessels; we investigated its role in the response of hematopoietic stem cells (HSCs) to liver fibrosis in mice.

**Methods:**

Purified (c-kit+/sca1+/lin-) HSCs were infused repeatedly into mice undergoing fibrotic liver injury. Chronic liver injury was induced in BoyJ mice by injection of carbon tetrachloride (CCl_4_) or placement on a methionine-choline–deficient diet. Some mice were irradiated and given transplants of bone marrow cells from C57BL6 mice, with or without the S1P antagonist FTY720; we then studied HSC mobilization and localization. Migration of HSC lines was quantified in Transwell assays. Levels of S1P in liver, bone marrow, and lymph fluid were measured using an enzyme-linked immunosorbent assay. Liver tissues were collected and analyzed by immunohistochemical quantitative polymerase chain reaction and sphingosine kinase activity assays. We performed quantitative polymerase chain reaction analyses of the expression of sphingosine kinase 1 and 2, sphingosine-1-phosphate lyase 1, and sphingosine-1-phosphate phosphatase 1 in normal human liver and cirrhotic liver from patients with alcohol-related liver disease (n = 6).

**Results:**

Infusions of HSCs into mice with liver injury reduced liver scarring based on picrosirius red staining (49.7% reduction in mice given HSCs vs control mice; *P* < .001), and hepatic hydroxyproline content (328 mg/g in mice given HSCs vs 428 mg/g in control mice; *P* < .01). HSC infusion also reduced hepatic expression of α-smooth muscle actin (0.19 ± 0.007-fold compared with controls; *P* < .0001) and collagen type I α 1 chain (0.29 ± 0.17-fold compared with controls; *P* < .0001). These antifibrotic effects were maintained with infusion of lymphoid progenitors that lack myeloid potential and were associated with increased numbers of recipient neutrophils and macrophages in liver. In studies of HSC cell lines, we found HSCs to recruit monocytes, and this process to require C-C motif chemokine receptor 2. In fibrotic liver tissue from mice and patients, hepatic S1P levels increased owing to increased hepatic sphingosine kinase-1 expression, which contributed to a reduced liver:lymph S1P gradient and limited HSC egress from the liver. Mice given the S1P antagonist (FTY720) with HSCs had increased hepatic retention of HSCs (1697 ± 247 cells in mice given FTY720 vs 982 ± 110 cells in controls; *P* < .05), and further reductions in fibrosis.

**Conclusions:**

In studies of mice with chronic liver injury, we showed the antifibrotic effects of repeated infusions of purified HSCs. We found that HSCs promote recruitment of endogenous macrophages and neutrophils. Strategies to reduce SIP signaling and increase retention of HSCs in the liver could increase their antifibrotic activities and be developed for treatment of patients with liver fibrosis.

**Editor's Notes**

**Background and Context**

There are no proven anti-fibrotic therapies in liver disease. Haematopoietic stem cells (HSC) have been suggested as being potentially efficacious in this setting, although this has not been well studied.

**New Findings**

Repeated injections of purified HSC markedly reduced liver fibrosis and improved liver function. This effect was increased by the co-administration of FTY720, a partial S1P receptor agonist, which enhanced their retention inside the liver.

**Limitations**

This study, while suggesting that HSC mediate their anti-fibrotic effect through recruitment of endogenous monocytes and neutrophils, does not conclusively identify the mechanisms mediating their anti-fibrotic effect.

**Impact**

There is a need for new therapies for patients with chronic liver disease, and this study demonstrates the therapeutic potential of treatment with HSC.

The incidence of chronic liver disease is increasing worldwide^1^ and is characterized by the progression of liver injury from hepatic fibrosis to cirrhosis, resulting in death from liver failure, complications of portal hypertension, or hepatocellular carcinoma.^2^ At present, liver transplantation remains the only curative treatment for end-stage liver disease but is limited by the availability of donor organs and the risks of lifelong immunosuppression.^3^, ^4^, ^5^ The development and resolution of hepatic fibrosis is recognized as a bidirectional process, with resolution of fibrosis mediated through degradation of hepatic collagen^6^ and apoptosis of activated hepatic myofibroblasts.^7^

Initial observations that bone marrow cells may contribute to hepatic repair and regeneration^8^, ^9^, ^10^ were followed by studies in animal models of chronic liver injury showing variable therapeutic effects.^11^, ^12^, ^13^, ^14^ Despite these mixed outcomes, multiple clinical studies of bone marrow (BM)-derived stem cell therapy already have been performed.^15^, ^16^, ^17^ The design of these studies has not permitted any meaningful conclusions and larger randomized controlled trials are underway.

The heterogeneous nature of the bone marrow cell populations studied to date has limited our understanding of their beneficial effects and prevented elucidation of potential mechanisms. Hematopoietic stem cells (HSCs) reside with the bone marrow niche, provide continual renewal and replacement of all blood cell lineages,^18^ and can be isolated routinely using cell surface markers.^19^ The continual homeostatic recirculation of bone marrow stem cells recently was described and the bioactive sphingolipid sphingosine 1-phosphate (S1P) was identified as a key regulator of this process.^20^ An S1P concentration gradient between body compartments is established by varying tissue distribution of sphingosine kinase (SPHK1/2) and sphingosine-1-phosphate lyase (SGPL)/sphingosine-1-phosphate phosphatase (SGPP),^21^, ^22^ and this gradient regulates the egress of HSCs from peripheral tissue into draining lymphatics. Binding of FTY720, a functional antagonist, to S1P receptors results in internalization and ubiquitin-dependent degradation without downstream signaling, thus rendering cells unresponsive to S1P.^23^ This blocks egress of HSCs, resulting in an accumulation of HSCs within peripheral tissues.^20^

We studied the effect of chronic liver injury on HSC mobilization and recruitment to the liver and showed reduced hepatic fibrosis after repeated therapeutic administration of a purified population of HSCs. Furthermore, we observed changes in S1P expression in chronic liver injury and showed prolonged hepatic retention of HSCs after administration of FTY720, which resulted in a further significant reduction in liver fibrosis when administered in conjunction with c-kit^+^, sca-1^+^ and lineage^neg^ (KSL) cell injections.

## Materials and Methods

### Murine Models of Liver Injury

Mice were housed in a temperature-controlled sterile animal facility with 12-hour light/dark cycles and free access to food and water. All experiments were conducted in accordance with the University of Birmingham ethics policy and the UK Animals (Scientific Procedures) Act 1986 (project license PPL 40/3201). C57/BL6 mice were obtained from Charles River Laboratories (London, UK) and BoyJ mice were obtained from a colony maintained at the Biomedical Services Unit of the University of Birmingham. To induce chronic liver injury, carbon tetrachloride (CCl_4_) (1 mg/kg diluted 1:4 in mineral oil; Sigma, London, UK) was injected twice weekly for 9 weeks, with control mice receiving mineral oil vehicle only. In separate experiments mice were fed either standard chow or a methionine-choline–deficient diet (MP Biomedical, London, UK) for 6 weeks.

### Experimental Protocols

Six- to 8-week-old female BoyJ mice received twice-weekly intraperitoneal injection of CCl_4_ (1 mg/kg in mineral oil) for 6 weeks, and then were allocated randomly to receive either purified cells isolated from 6- to 8-week-old male C57BL6 mice as described, or no treatment. Cell injections were administered on the first day of weeks 7, 8, and 9 of liver injury, and mice were killed 1 week after the final cell injection and 72 hours after the final CCl_4_ injection. In some experiments FTY720 (Cayman Chemicals, Cambridge, UK; 1 mg/kg in phosphate-buffered saline + 0.1% dimethyl sulfoxide) or vehicle control was administered by intraperitoneal injection 3 times per week from week 6 until death.

### Bone Marrow Transplantation

Six-week-old C57/BL6 mice received lethal irradiation (9 Gy in 2 divided doses) followed by transplantation via tail vein injection of 1 × 10^8^ whole bone marrow cells isolated from 6-week-old BoyJ mice. After 4 weeks, mice were allocated randomly to receive either twice-weekly injection of CCl_4_ (1 mg/kg) or mineral oil vehicle, for 8 weeks followed by death.

### Immunohistochemistry

Picrosirius red (PSR) staining was performed using 0.1% Direct Red 80 (Sigma) in saturated picric acid according to standard protocols. Quantification of PSR and α-smooth muscle actin (α-SMA) staining was performed by threshold analysis of 10 nonoverlapping randomly selected fields of view per slide at a magnification of 20× using ImageJ software (National Institutes of Health, Bethesda, MD), and was expressed as the percentage of positive staining of the total area. F4/80 and Ly6G staining was quantified by counting individual positive cells in 6 nonoverlapping randomly selected fields of view per slide at magnifications of 20× and 100× and expressed as cells per field of view.

### S1P and SphK Quantification

SphK activity was determined using the Sphingosine Kinase Activity Assay (K-3500; Echelon, Le-Perray-en-Yvelines, France) according to the manufacturer’s instructions. A standard curve was created and results were normalized to the protein content of the sample and time (pmol S1P/mg protein/min). S1P concentrations were measured using the sphingosine-1-phosphate enzyme-linked immunosorbent assay kit (K-1900; Echelon) according to the manufacturer’s instructions, S1P concentrations in serum and lymph fluid were expressed as molar quantities and in liver and bone marrow were normalized to the protein content of the sample (pmol S1P/mg protein).

### Chemotaxis Assay

A total of 600 μL Stem Pro 34 serum-free media alone or containing the relevant concentration of S1P (Cayman Chemicals) was placed in individual wells of a 12-well plate (Corning, London, UK) and 6.5-mm Transwell inserts (5-μm pore diameter) placed in each well. A total of 100 μL of cell suspension (1 × 10^6^ cells/mL) was added to the insert and incubated for 3 hours. Cell migration was assessed by quantification of the number of cells in each lower well using flow cytometry as described; migration was expressed as a percentage of the input cells in the lower well. S1P and FTY702-P were prepared by dissolving in 95% dimethyl sulfoxide/5% 1 N HCl (Sigma) and diluted for use in phosphate-buffered saline + 3% fatty acid free bovine serum albumin (Sigma). W146 (Sigma) was dissolved in methanol containing 0.05% acetic acid and diluted for use in cell culture media.

### Cell Culture

Studies of HSC trafficking have been limited by difficulties in isolating a sufficient number of cells, in some experiments in this study we used an immortalized HSC line (hematopoietic progenitor cell line 7 [HPC-7]), which expresses common murine HSC markers and transcription factors,^24^ which have been used in previous studies of HSC recruitment.^25^ HPC-7 cells were cultured in StemPro Serum Free Media 34 (Invitrogen, London, UK) supplemented with penicillin, streptomycin, glutamine, and 100 ng/mL recombinant murine Stem Cell Factor (Invitrogen). For colony-forming unit assays cells were added to MethoCult GF media (Stem Cell Technologies, Cambridge, UK) and incubated for 10 days, and the total number of myeloid colonies per assay were determined by counting under low-power magnification.

### Statistics

Statistical analyses were performed using GraphPad (La Jolla, CA) Prism version 5.0. Differences between groups were analyzed using either the 2-tailed unpaired Student *t* test or multiple group comparisons with 1-way analysis of variance with Bonferroni post-test correction unless otherwise stated. A result was considered significant when the *P* value was less than .05.

## Results

### Bone Marrow–Derived HSCs Are Mobilized and Recruited to the Liver During Chronic Liver Injury

**Figure 1:**
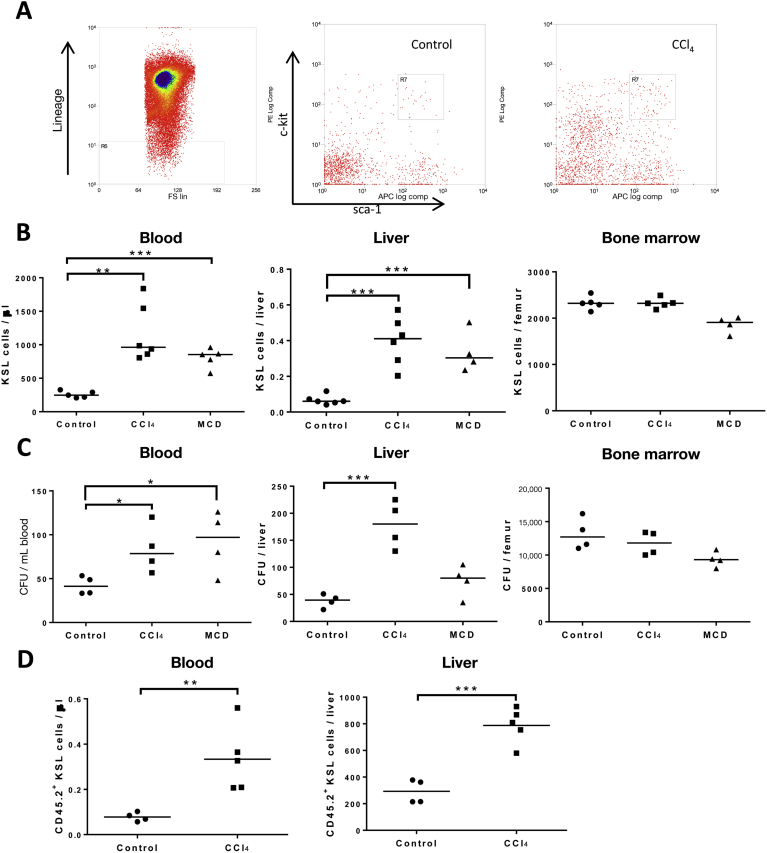
Liver injury increases mobilization and recruitment of HSCs to the liver. (*A*) KSL cells were quantified by flow cytometry and representative plots are shown. (*B*) Numbers of KSL cells were quantified in the peripheral blood, livers, and BM of CCl_4_-, methionine-choline–deficient (MCD), and mineral oil–treated mice. Data from individual mice are shown with means indicated by the *horizontal line* (n = 6 per group). (C) Colony-forming potential of cells isolated from liver, blood, and bone marrow were quantified in myeloid colony-forming unit (CFU) assays. Data from individual mice are shown with means indicated by the *horizontal line* (n = 6 per group). (*D*) BoyJ (CD45.1) mice were irradiated lethally (9 Gy in 2 divided doses) and received unfractionated BM from C57Bl/6 (CD45.2) mice followed by administration of CCl_4_ or mineral oil for 8 weeks. Higher numbers of bone marrow–derived CD45.2^+^ KSL were found in the blood and livers of mice after CCl_4_ liver injury (n = 6 per group). *P < .05, **P < .01, and ***P < .001.

**Supplementary Figure 1:**
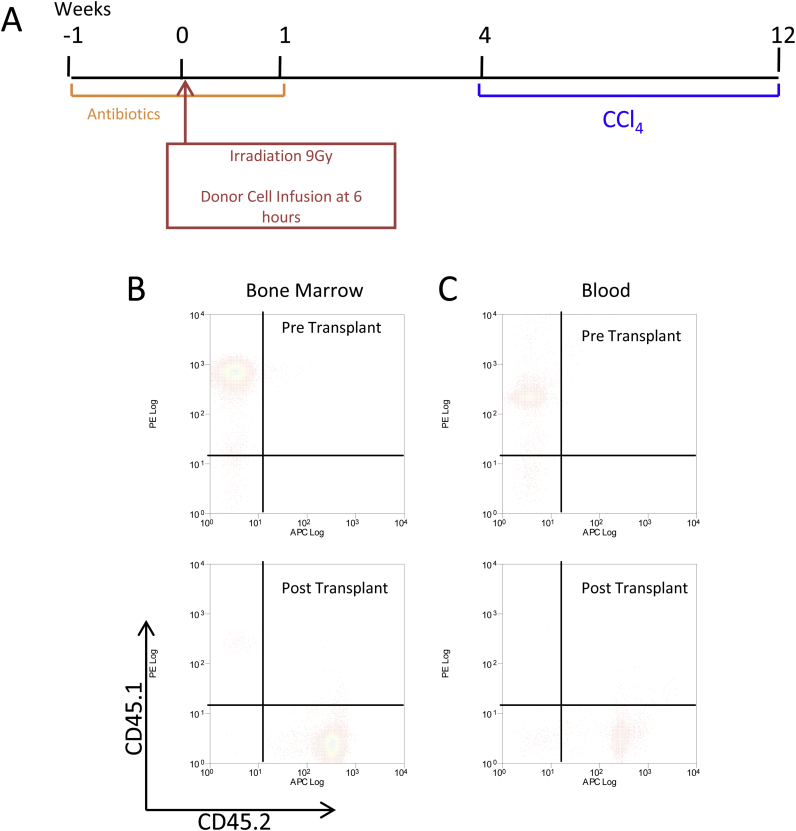
Bone marrow transplantation studies. (*A*) Recipient BoyJ (CD45.1) mice received Baytril (Bayer Corporation, Dallas, TX) antibiotic in drinking water 1 week before and 1 week after irradiation with 9 Gy in 2 divided doses on the same day. Bone marrow cells were obtained from donor C57BL6 (CD45.2) mice by flushing of the tibia and femurs. Six hours after irradiation donor bone marrow cells were infused into recipient mice by tail vein injection. After a 4-week recovery, transplanted mice were divided into 2 groups at random with 1 group receiving twice-weekly intraperitoneal administration of carbon tetrachloride (1 mg/kg) in mineral oil and the other group receiving intraperitoneal injections of mineral oil alone for a further 8 weeks. (*B* and *C*) Samples of bone marrow and peripheral blood were treated with red cell lysis buffer and labeled with fluorescent antibodies to CD45.1 and CD45.2. Analysis of CD45 expression by flow cytometry showed almost complete CD45.2 expression after transplantation compared with CD45.1 expression before transplantation. Representative flow cytometry plots of multiple experiments are shown.

The effect of liver injury on the mobilization and recruitment of BM-derived HSCs was investigated in the model of CCl_4_-induced liver injury. Higher numbers of HSCs (KSL), were isolated from the peripheral blood (0.397 ± 0.05 vs 0.065 ± 0.07 KSL cells/μL blood; *P* < .001) and liver (1163 ± 173 vs 258.5 ± 22 KSL cells/liver; *P* < .01) of mice with a CCl_4_ injury compared with control mice (Figure 1*B*). There also were marked increases in colony-forming unit potential from cells isolated from liver and peripheral blood, but not BM, in CCl_4_ injury (Figure 1*C*). Similar results were seen in the setting of methionine-choline–deficient diet–induced liver injury. Liver resident populations of HSCs have been described (Taniguchi et al 1996^26^) and further studies to confirm the bone marrow origin of the isolated KSL cells were performed. Bone marrow chimerism was established (Supplementary Figure 1) and donor BM-derived HSCs were identified as CD45.2^+^ KSL. Significant increases in CD45.2^+^ KSL cells were observed within the liver (788.2 ± 59 vs 292.6 ± 45; *P* < .01) and peripheral blood (0.333 ± 0.06 vs 0.077 ± 0.01; *P* < .01) of CCl_4_-injured mice (Figure 1*D*), whereas BM populations remained constant. Given the mobilization and hepatic recruitment of HSCs in liver injury the potential therapeutic benefits of administering purified HSCs was investigated.

### Infused KSL Cells Reduce Fibrosis in the CCl_4_ Model of Liver Injury

**Figure 2:**
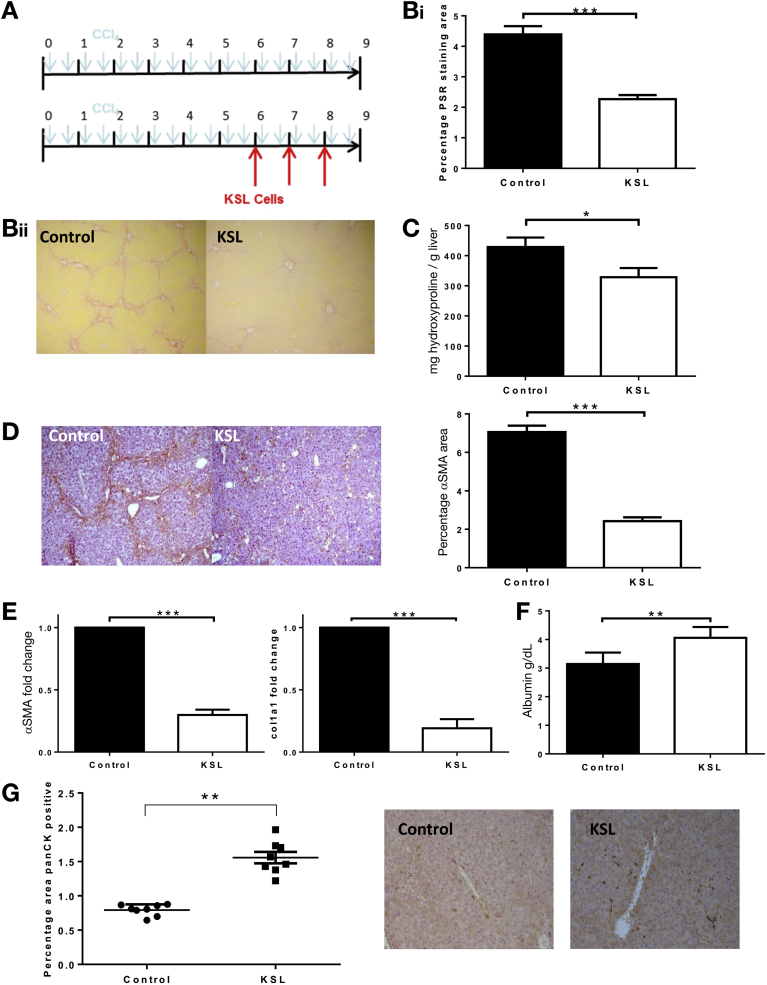
Repeated injection of HSC KSL reduced hepatic fibrosis in a model of CCl_4_ liver injury. (*A*) Liver injury was induced in 8-week-old C57/Bl6 mice by 8 weeks of twice-weekly intraperitoneal injections of CCl_4_. Mice were divided into 2 groups of 8 and 1 group received injections of 5 × 10^4^ KSL cells via tail vein at weeks 6, 7, and 8. Representative photomicrograph images of PSR of livers show a reduction in collagen staining of livers from KSL-treated mice, with loss of bridging fibrosis, compared with untreated control mice (magnification, 40×). Six random nonoverlapping images were obtained from each section and staining was quantified as a percentage of the image positive for PSR and α-SMA using ImageJ software. (*B*) Quantitative analysis of picrosirius red staining and (*C*) biochemical measurement of hepatic hydroxyproline content verified the reduction in hepatic fibrosis after KSL injections. The number of activated hepatic stellate cells, as indicated by (*D*) representative photomicrograph images of α-SMA staining, was reduced after KSL injections, which was confirmed by morphometric analysis of hepatic α-SMA staining and by (*E*) hepatic gene expression of α-SMA and col1a1. (*F*) Serum albumin levels were higher in mice receiving KSL injections. (*G*) Hepatic oval cell numbers, as indicated by Pan-CK were increased after KSL cell administration. Data are from n = 8 per group and 3 independent experiments. *P < .05, **P < .01, and ***P < .001 vs control.

**Supplementary Figure 2:**
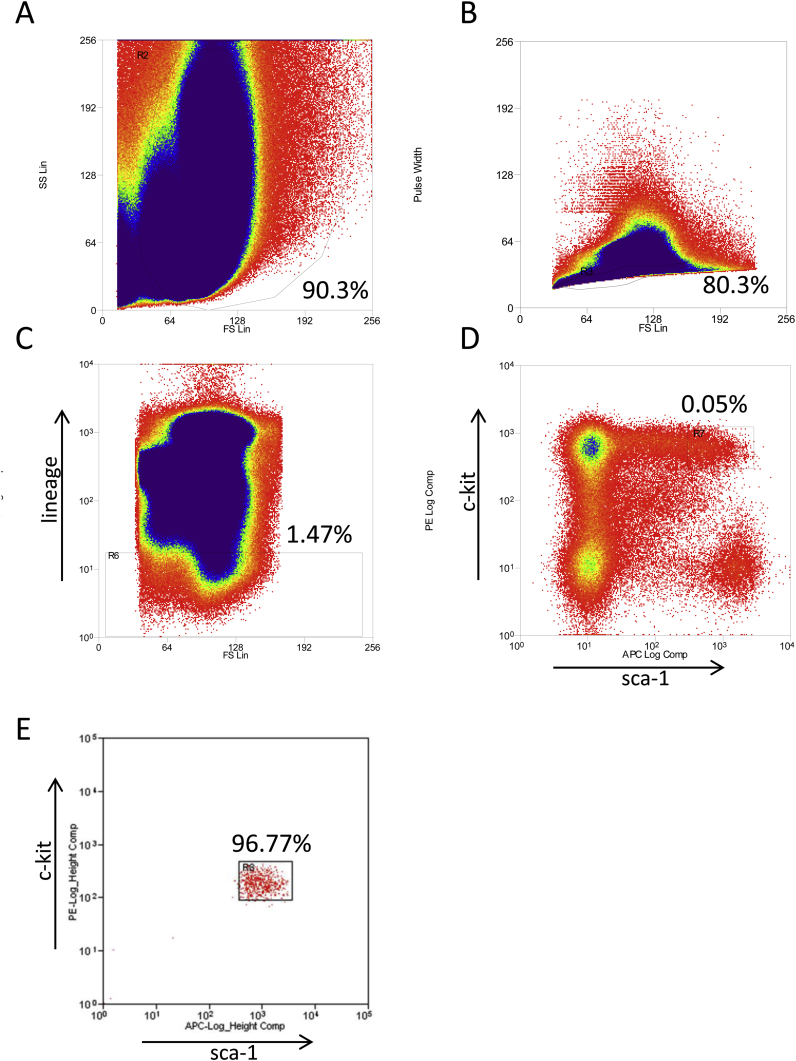
Isolation of KSL cells. Bone marrow cells were obtained from donor mice after dissection of the tibia and femurs by flushing the marrow cavity with Dulbecco's modified Eagle medium + 10% fetal calf serum. Cells were treated with red cell lysis buffer and filtered before labeling with fluorescently conjugated antibodies to lineage specific markers (CD11b, CD8a, CD5, Ter119, Gr-1, and B220), c-kit, and sca-1. Cells were isolated by (*A*) initially excluding dead cells and debris on a forward scatter–side scatter plot and (*B*) excluding doublets of cells on a forward scatter–pulse width plot. (*C*) The lineage-negative population was determined and (*D*) plotted according to the expression of c-kit and sca-1 surface markers. HSCs were defined as KSL and represented 0.05% of the total bone marrow cell population. (*E*) The purity of the isolated cells then was checked by determining lineage marker, c-kit, and sca-1 expression on a sample of isolated cells.

KSL HSCs were isolated from donor bone marrow and purity of more than 96% was confirmed in all experiments (Supplementary Figure 2). Repeated injections of KSL cells (Figure 2*A*) resulted in a 49.7% reduction in hepatic fibrosis determined by PSR quantification (2.21% ± 0.12% vs 4.38% ± 0.27% staining; *P* < .0001) (Figure 2*B*), and by reduced hepatic hydroxyproline content (328.5 ± 30.4 vs 428.4 ± 31.9 μg/g liver; *P* < .05) (Figure 2*C*). Resolution of fibrosis is dependent on apoptosis of α-SMA^+^ myofibroblasts within the liver, and α-SMA staining was 65.6% lower after KSL cell injections (2.42 ± 0.2 vs 7.05 ± 0.33; *P* < .0001) (Figure 2*D*) and was associated with down-regulation of hepatic α-SMA (0.19 ± 0.007-fold vs control; *P* < .0001) and col1a1 (0.29 ± 0.17-fold vs control; *P* < .0001) gene expression (Figure 2*E*). Serum albumin level was higher in treated mice than in untreated controls (4.06 ± 0.37 vs 3.14 ± 0.39; *P* < .01) (Figure 2*F*). There also were increases in hepatic oval cell numbers (Figure 2*G*).

### Antifibrotic Effect of KSL Cells Is Associated With Enhancement of Endogenous Repair Mechanisms

**Figure 3:**
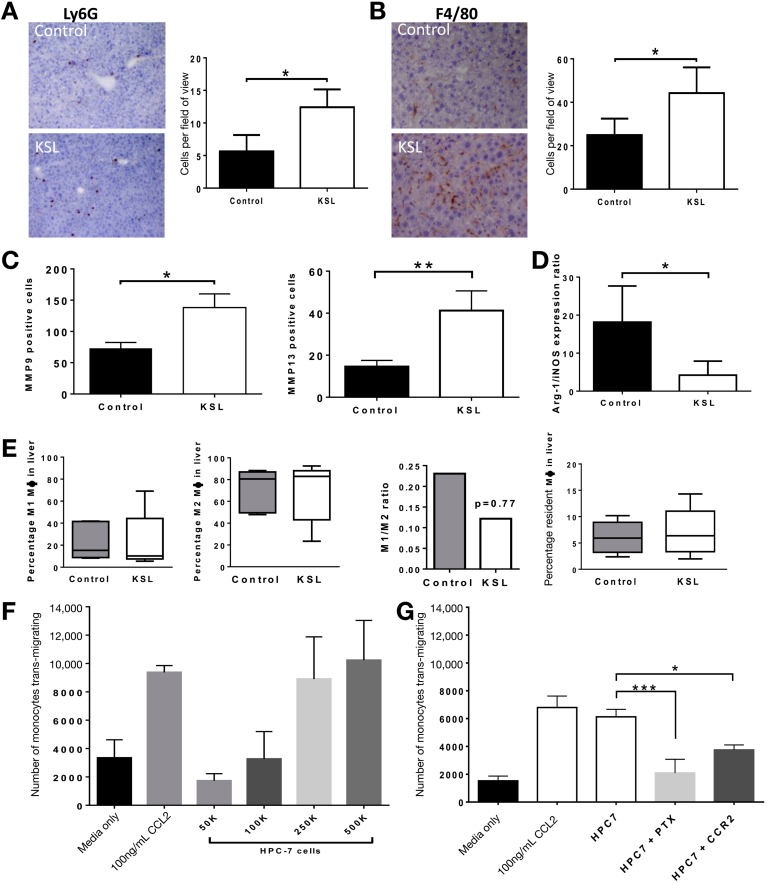
Impact of administered HSCs on endogenous cell recruitment and expansion in the injured liver. There was marked expansion in the number of (*A*) recipient-derived Ly6G and (*B*) F4/80-immunopositive cells in the livers of mice after KSL injection as indicated in representative photomicrographs. (*A*) Ly6G and (*B*) F4/80 cells were counted manually in 6 random nonoverlapping fields of view (×100 F4/80, ×20 Ly6G). (*C*) MMP9 and MMP13 expression in the liver was seen to increase after KSL cell administration, in association with a (*D*) reduced Arg-1/iNOS expression ratio. (*E*) Flow cytometric analysis of the digested livers (gating strategy is described in the Supplementary Materials and Methods section) characterized the macrophage subsets within the liver. (*F*) Murine monocytes were seen to transmigrate toward CCL2 and HPC-7 HSCs in a dose-dependent fashion. (*G*) Migration of murine monocytes toward HPC-7 was chemokine-dependent and inhibited by neutralizing CCR2 antibody. Data are from n = 8 per group and 3 independent experiments, expressed as means ± SD of the number of cells per field of view shown or medians ± interquartile range. *P < .05, **P < .01, and ∗∗∗*P* < .001.

**Supplementary Figure 3:**
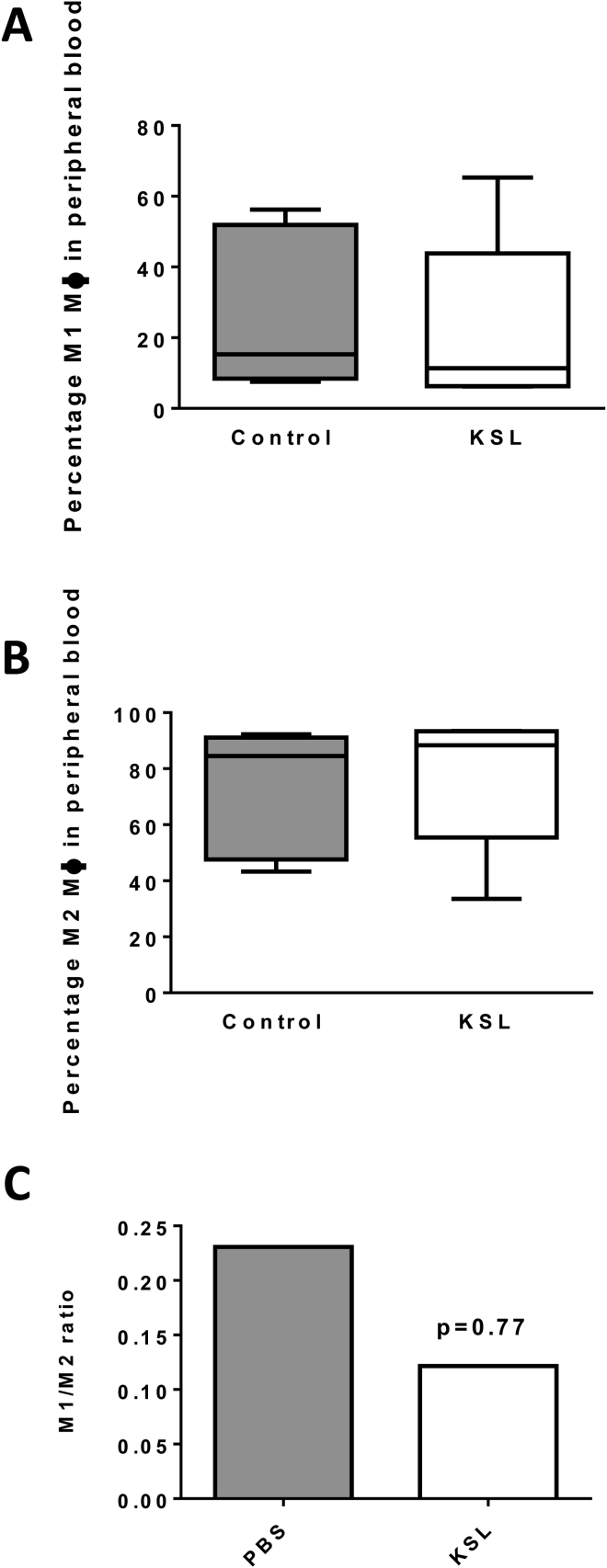
(*A* – *C*) Flow cytometric analysis of peripheral blood was performed to characterize the macrophage subsets (M1/M2) and their ratio. The following gating strategy was used: M1-macrophages (gated CD45^+^CD3^-^CD11b^+^F4/80^+^Ly-6G^-^Ly6C^hi^) and M2-macrophages (gated CD45^+^CD3^-^CD11b^+^F4/80^+^Ly-6G^-^Ly6-C^lo^). Data are expressed as median ± interquartile range. PBS, phosphate-buffered saline.

The fate of injected cells was investigated, but donor-derived CD45.2^+^ cells could not be detected in significant numbers within the liver 7 days after injection. Quantification of cell populations within the liver showed a 219% increase in neutrophils (12.4 ± 2.7 vs 5.646 ± 2.51 Ly6G^+^ cells per field of view; *P* < .0001) and a 177% increase in macrophages (44.2 ± 11.8 vs 24.99 ± 7.5 F4/80^+^ cells per field of view; *P* < .0001) after KSL injections (Figure 3*A* and *B*). The absence of CD45.2 staining indicated that these increases were caused by increases in endogenous cell populations rather than differentiation of injected KSL. Although there were increases in matrix metalloproteinase (MMP)9- and MMP13-expressing cells (Figure 3*C*), and a reduction in the Arg-1/iNos ratio (Figure 3*C*), in the livers of mice receiving KSL infusions there were no differences in macrophage subsets within the liver (Figure 3*E*) or blood (Supplementary Figure 3*A–C*) after KSL cell infusion. The ratio of Ly-6C^hi^/Ly-6C^lo^ (M1-like to M2-like) macrophages was not different between the control (0.23) and KSL groups (0.12). Murine monocytes migrated toward HPC-7 cells in a dose-dependent fashion and at similar levels to chemokine (C-C motif) ligand 2 (Figure 3*F*), a classic monocyte chemoattractant. This migration was chemokine dependent, as shown by blockade seen after pertussis toxin administration (Figure 3*G*). Notably, the majority of this reduction was achieved when a CCR2-blocking antibody was used (Figure 3*G*).

### Antifibrotic Effect of KSL Cells Occurs Irrespective of Their Myeloid Differentiation Potential

**Figure 4:**
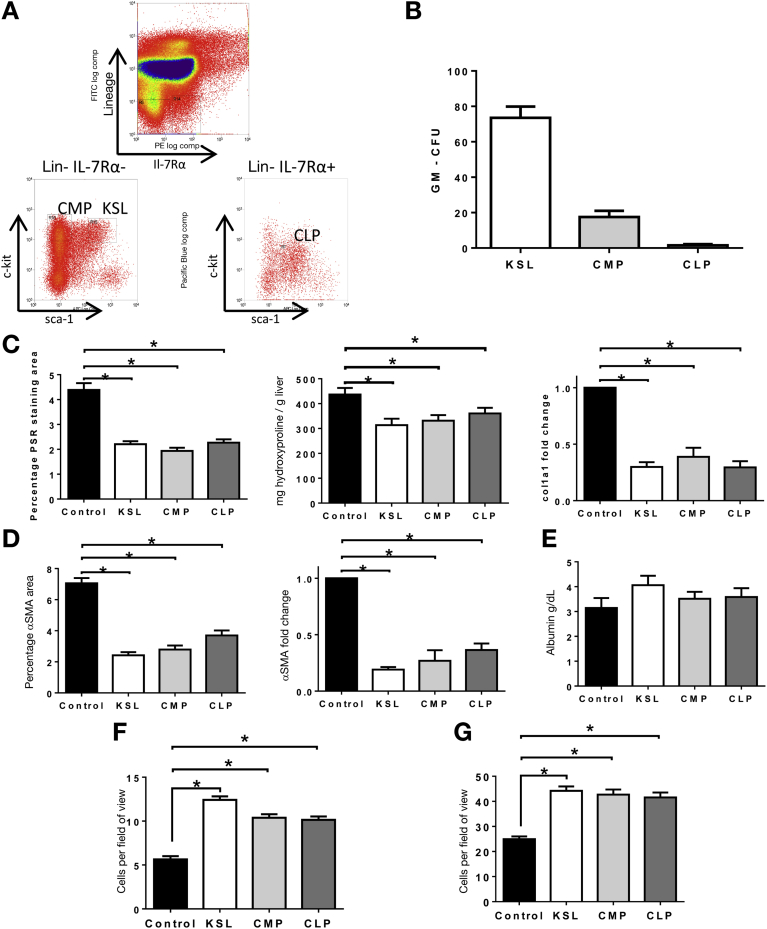
Committed HSC subsets without myeloid properties also are able to reduce liver fibrosis. (*A*) Representative plots of common myeloid progenitors and common lymphoid progenitors were isolated using surface antigen expression (CMP, interleukin [IL]7Ra^-^ c-kit^+^ sca1^+^ lineage^-^; CLP, interleukin [IL]7Ra^+^ c-kit^lo^ sca1^lo^ lineage^-^). (*B*) KSL and CMP were able to form myeloid colonies in vitro, whereas CLP lacked this ability. Data are expressed as the mean number of colonies per 500 plated cells shown (±SD) from 3 separate isolations. (*C*) Tail vein injections of CMP or CLP cells at weeks 6, 7, and 8 of CCl_4_ injury resulted in a similar reduction in hepatic fibrosis to that seen with KSL injections as assessed by quantitative analysis of picrosirius red staining, biochemical measurement of hepatic hydroxyproline content, and hepatic gene expression for col1a1. (*D*) Similarly, CMP and CLP injections also reduced the number of activated hepatic stellate cells to a level seen with KSL injections, as indicated by morphometric analysis of hepatic α-SMA staining and by hepatic gene expression of α-SMA. (*E*) Serum albumin levels also were increased after CMP and CLP injections. There were increased numbers of (*F*) endogenous Ly6G neutrophils and (*G*) F4/80 macrophages in the liver after CMP and CLP infusions. Data are from n = 8 per group and 3 independent experiments. *P < .05 vs control.

To explicitly establish whether antifibrotic effects of KSL were mediated through differentiation to macrophages, the effect of injecting committed myeloid or lymphoid progenitor cells was determined (Figure 4*A*). Common myeloid progenitors (CMPs) established myeloid colonies whereas common lymphoid progenitors (CLP) did not (Figure 4*B*). Repeated injections of either progenitor population resulted in reduced hepatic fibrosis (Figure 4*C* and *D*), including a reduction in PSR staining (CMP, 1.94% ± 0.29%; CLP, 2.27% ± 0.13%; vs control, 4.38% ± 0.27% staining; *P* < .01 both vs control) (Figure 4*C*) and a reduction in α-SMA staining (CMP, 2.79% ± 0.25%; CLP, 3.69% ± 0.33%; vs control 7.05% ± 0.39% staining; *P* < .05 both vs control) (Figure 4*D*). This antifibrotic effect was similar to that observed with injections of KSL and confirmed that myeloid differentiation was not required to mediate this effect. As with KSL cells, infusions of CMP and CLP cells also were associated with histologic evidence of increased numbers of endogenous Ly6G neutrophils and F4/80 macrophages (Figure 4*F* and *G*) in the liver.

### Hepatic Sphingosine-1-Phosphate Expression and Activity Increases in Rodent and Clinical Liver Injury

**Figure 5:**
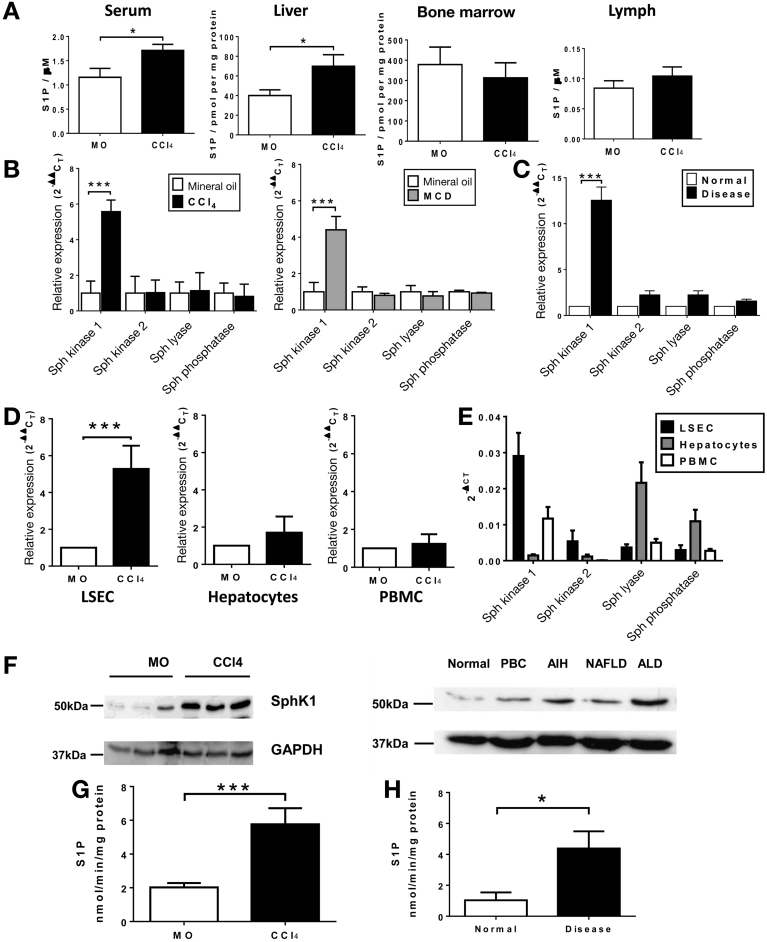
Regulation of S1P levels in murine and human chronic liver injury. (*A*) Eight-week-old C57/BL6 mice received CCl_4_ or mineral oil (MO) by intraperitoneal injection twice weekly for 8 weeks. S1P levels were higher in the livers and sera of mice after 8 weeks of CCl_4_ liver injury compared with MO controls, but were unchanged in bone marrow and lymph (n = 5 per group). (*B*) Hepatic expression, by quantitative polymerase chain reaction, of SphK1 was increased, but there were no changes in gene expression for other enzymes (SphK2, SGPL1, and SGPP1) involved in S1P regulation (n = 6 per group). A similar pattern of gene expression was observed in mice fed a methionine-choline deficient (MCD) diet, an alternative model of chronic liver injury (n = 6 per group). (*C*) Hepatic gene expression of SphK1, SphK2, SGPL1, and SGPP1 were quantified in normal human liver and also human cirrhotic liver from patients with alcohol-related liver disease (n = 6 per group). (*D*) Analysis of cell populations from chronically injured murine livers showed up-regulation of SphK1 gene expression in hepatic sinusoidal endothelial cells (LSEC), but not hepatocytes or peripheral blood mononuclear cells when compared with MO controls (n = 3 per group, 3 independent experiments). Gene expression also was studied in constituent cells from explanted human cirrhotic livers and associated peripheral blood mononuclear cells (n = 6 per group). (*E*) Represents adherent cells per field of view as assessed by intravital microscopy. (*F*) There was an increase in SphK1 protein expression in the CCl_4_-treated mice by Western blot (representative of 3 independent experiments). Samples of liver tissue were used in an adenosine triphosphate depletion assay to measure the enzymatic activity of SphK in injured and normal liver tissue (*bottom left panel*). (*G*) The rate of phosphorylation of sphingosine to S1P was higher in CCl_4_-injured mice, as determined from the rate of depletion of adenosine triphosphate and expressed as nmol of S1P produced per minute per mg of liver protein (n = 5 per group). (*F*) Samples of human liver tissue were lysed for protein extraction and expression of SphK1 and glyceraldehyde-3-phosphate dehydrogenase (GAPDH) by Western blot (representative of 3 independent experiments) showed an increase in all cirrhotic liver samples (*top right panel*). (*H*) The rate of phosphorylation of Sphingosine to S1P was higher in human chronic liver injury, as determined from the rate of depletion of ATP and expressed as nmol of S1P produced per minute per mg of liver protein (n = 5 per group). *P < .05, ***P < .001 vs control. AIH, autoimmune hepatitis; ALD, alcoholic liver disease; NAFLD, nonalcoholic fatty liver disease; PBMC, peripheral blood mononuclear cell.

**Supplementary Figure 4:**
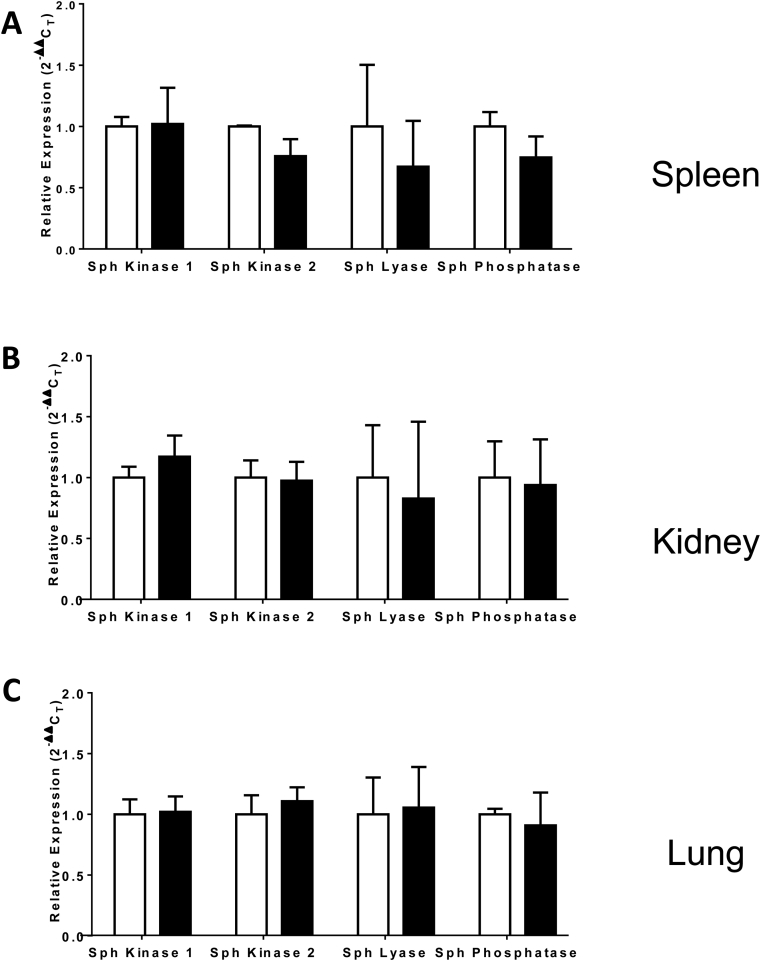
S1P gene expression in extrahepatic organs. Chronic liver injury was induced in age- and sex-matched C57/BL6 mice by twice-weekly intraperitoneal administration of carbon tetrachloride (1 mg/kg) in mineral oil for 8 weeks. Control mice received twice-weekly intraperitoneal injections of mineral oil. Mice were killed and gene expression of sphingosine kinase 1, sphingosine kinase 2, sphingosine lyase, and sphingosine phosphatase relative to glyceraldehyde-3-phosphate dehydrogenase was determined in (*A*) spleen, (*B*) kidney, and (*C*) lung. *Bars* represent means ± SEM fold change relative to control mice. N = 5 per group.

**Supplementary Figure 5:**
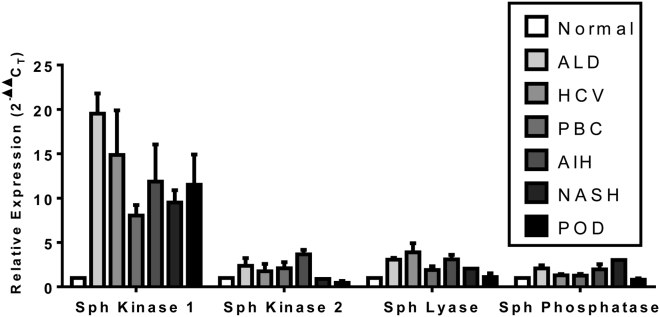
Hepatic gene expression across differing etiologies of liver disease. Samples of frozen liver tissue were obtained from either normal donor liver or explanted livers from patients with liver disease. Gene expression of sphingosine kinase 1, sphingosine kinase 2, sphingosine lyase, and sphingosine phosphatase relative to glyceraldehyde-3-phosphate dehydrogenase was determined. *Bars* represent means ± SEM fold change relative to normal liver (no statistical comparison, n = 3 per group). AIH, autoimmune hepatitis; ALD, alcoholic liver disease; HCV, hepatitis C virus; NAFLD, nonalcoholic fatty liver disease; PBC, primary biliary cirrhosis; POD, paracetamol overdose.

Because S1P, mediated by differences in concentration gradients, regulates the homeostatic trafficking of HSCs between tissue compartments, we determined changes in liver injury. In CCl_4_ injury S1P levels were 1.7-fold higher in the liver (68.79 ± 7.42 vs 39.45 ± 7.06 pmol per mg protein; *P* < .05) and 1.5-fold higher in the serum (1.71 ± 0.13 vs 1.15 ± 0.18 μmol/L; *P* < .01) with no significant change in BM or lymph concentrations (Figure 5*A*). To determine the factors influencing S1P levels we studied its cognate metabolizing enzymes. SphK1 phosphorylates sphingosine to produce S1P, and hepatic gene expression of SphK1 was up-regulated during murine CCl_4_ liver injury (5.57 ± 0.65-fold vs control; *P* < .0001), whereas expression of sphingosine kinase 2, SGPL, and SGPP remained unchanged (Figure 5*B*). Similar findings were observed in human chronic liver disease (Figure 5*C*). SphK1 up-regulation was not seen outside the murine liver (Supplementary Figure 4) and also was observed in mice fed the methionine-choline–deficient diet, an alternative model of chronic liver injury (Figure 5*B*). Analysis of isolated murine cell types showed significant up-regulation of SphK1 gene expression in liver sinusoidal endothelial cells during liver injury (5.26 ± 1.26-fold vs control; *P* < .001) without significant alteration in hepatocyte or peripheral blood mononuclear cell gene expression (Figure 5*D*). SphK1 expression was 12-fold higher in human subjects with chronic liver disease than in normal controls (12.55 ± 6.1-fold vs control; *P* < .01) (Figure 5*C*), and this observation held true for different etiologies of liver disease (Supplementary Figure 5). SphK1 was the most abundant enzyme in hepatic sinusoidal endothelial cells and peripheral blood mononuclear cells, whereas hepatocytes predominantly expressed SGPL and SGPP (Figure 5*E*). Increased SphK1 was detected by Western blot (Figure 5*F*) in both murine and human chronic liver injury, and SphK1 enzymatic activity was 2.7-fold higher (5.76 ± 0.95 vs 2.07 ± 0.25 nmol/min/mg protein; *P* < .01) (Figure 5*G*) in liver tissue from mice with CCl_4_ injury. SphK1 enzymatic activity also was increased in chronically diseased human liver tissue (Figure 5*H*).

### Blocking Migration to S1P with FTY720 Specifically Increases Retention of KSL Cells in the Injured Liver

**Figure 6:**
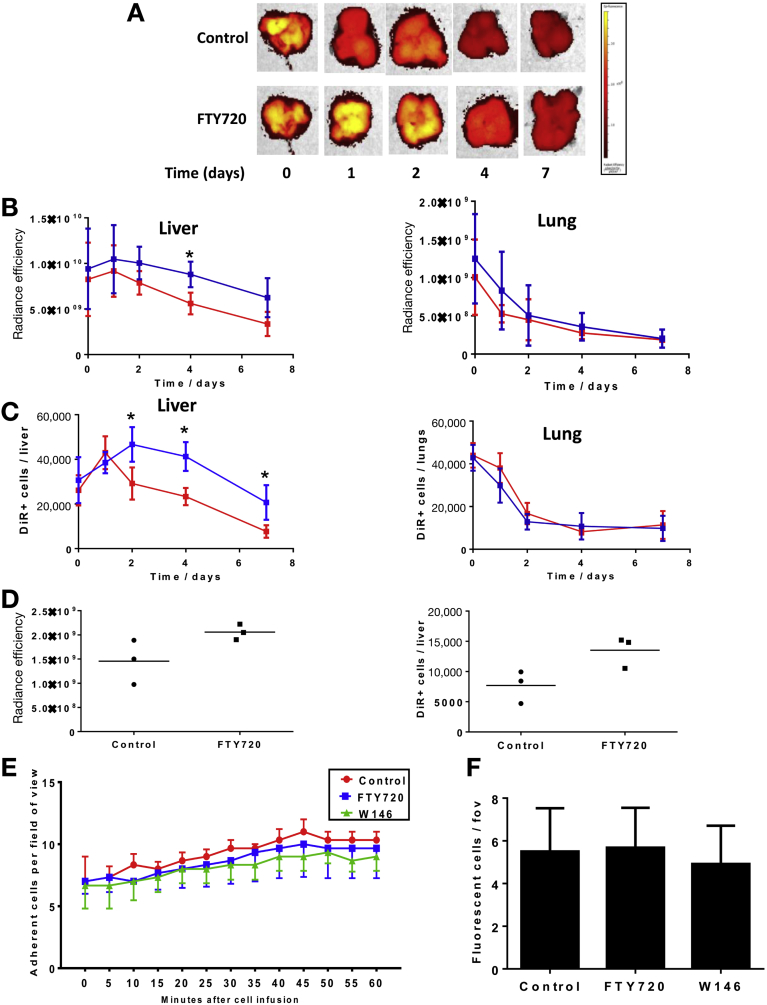
FTY720 treatment increased the number of injected HSCs within the liver. DiR-labeled HPC-7 cells were administered via tail vein to CCl_4_-injured mice that also were treated with either FTY720 (1 mg/kg) or vehicle control starting 1 day before cell injection and continuing for 7 days. (*A*) Representative combined photographic and fluorescence intensity images of livers from mice injected with HPC-7 cells treated with either FTY720 or vehicle at time points after cell injection. (*B*) The number of DiR-labeled HPC-7 cells within the liver and lungs then were quantified using fluorescence intensity by (*B*) in vivo imagine system (IVIS) and (*C*) flow cytometric analysis of digested organs up to 7 days after injection. Greater numbers of DiR-labeled cells were detected in the liver at days 2, 4, and 7 after injection in mice treated with FTY720 (*blue line*; n = 3 per group, 3 independent experiments). (*D*) Greater numbers of injected DiR-labeled KSL cells were present in the livers of mice treated with FTY720 (n = 3 per group) using both fluorescence intensity by IVIS (London, UK) and by flow cytometric analysis of digested liver (n = 3 per group, 3 independent experiments). (*E*) To establish if S1P has a role in engraftment of HSCs to the liver (as well as retention), CFSE-labeled HPC-7 were pretreated with FTY720, W146, or control media and injected into mice with CCl_4_ liver injury. Recruitment of HSCs to the liver was assessed in real time by intravital microscopy. Pretreatment of HPC-7 with FTY720 or W146 did not alter recruitment to injured liver for 60 minutes after injection (*P* = NS by 2-way analysis of variance with the Bonferroni multiple comparison test, n = 3 per group, 6 independent experiments). (*F*) After completion of intravital microscopy experiments, liver tissue sections were analyzed by fluorescent microscopy (fluorescein isothiocyanate filter). The number of individual fluorescent cells was counted manually in 6 random nonoverlapping fields of view (magnification, 20×) per section. No difference in the number of injected cells within the liver was seen between cells treated with FTY720-P, W146, or control media (n = 3 each group). *P < .05. fov, field of view.

**Supplementary Figure 6:**
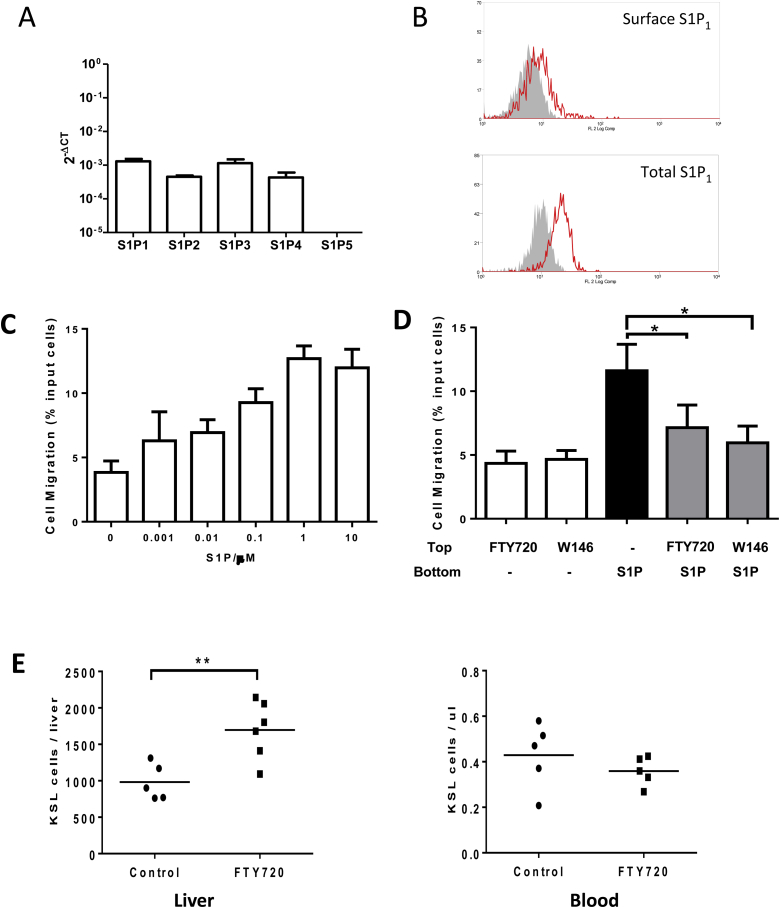
FTY720 alters HSC trafficking through modulation of S1P1. (*A*) Gene expression of S1P receptors 1–5 was determined in freshly isolated KSL cells relative to glyceraldehyde-3-phosphate dehydrogenase by the 2^-ΔCT^ method. *Bars* represent means ± SEM expression in 3 independent experiments. (*B*) Surface and total S1P1-receptor expression on freshly isolated KSL cells was analyzed by flow cytometry. Representative histograms of 3 independent experiments are shown (*grey area*, isotype control; *red line*, S1P1 antibody). (*C*) A total of 1 × 10^5^ KSL cells were added to the top well of a Transwell and migration to varying concentrations of S1P was assessed over a 4-hour period. Cells migrating into the bottom well were analyzed for expression of c-kit and sca-1 and quantified by flow cytometry. KSL cells migrating to the lower well were expressed as a percentage of the total number of KSL cells added to the top well. *Bars* represent the means ± SEM of 3 separate experiments. (*D*) S1P-receptor dependence of migration of KSL cells to S1P was assessed by placing cells in the upper chamber of a Transwell and quantifying their migration to the presence or absence of S1P in the lower chamber. Treatment of KSL cells with either FTY720 (S1P-receptor partial agonist) or W146 (S1P-receptor 1 antagonist) inhibited their migration to S1P in Transwell assays (3 independent experiments). (*E*) Administration of daily intraperitoneal FTY720 (1 mg/kg) for 7 days in CCl_4_-injured mice resulted in an increased number of KSL cells in the liver but circulating KSL cells remained unchanged (n = 6 per group; 2 independent experiments). *P < .05 and ∗∗*P* < .01.

**Supplementary Figure 7:**
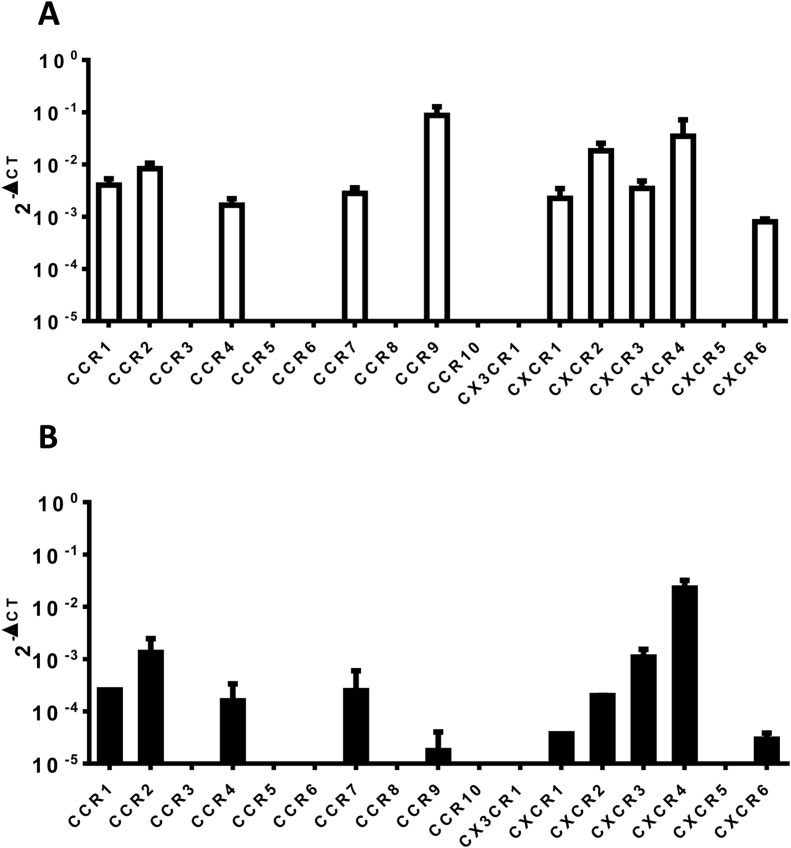
Chemokine receptor expression on KSL and HPC-7 cells. Gene expression of chemokine receptors in (*A*) freshly isolated KSL cells and (*B*) cultured HPC-7 cells was determined by quantitative polymerase chain reaction. Expression relative to glyceraldehyde-3-phosphate dehydrogenase by the 2^-ΔCT^ method was determined. Bars represent means ± SEM expression in 3 independent experiments.

**Supplementary Figure 8:**
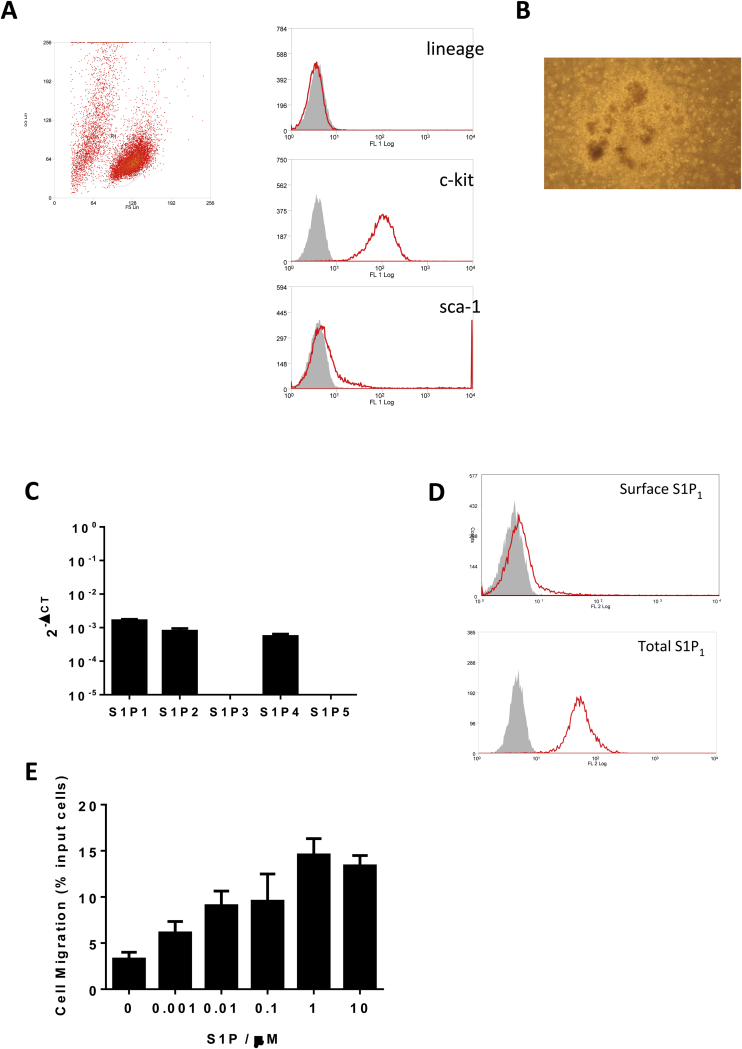
Characterization of HPC-7 cell line. (*A*) Cultured HPC-7 cells were labeled with fluorescently conjugated antibodies to lineage markers (CD11b, CD5, CD8a, Ter119, Gr-1, and B220), c-kit, and sca-1, and analyzed by flow cytometry. Dead cells and debris were excluded by gating on a plot of forward scatter–side scatter and surface expression of lineage markers, c-kit, and sca-1 were measured. Representative histograms are shown (isotype control, *grey area*; antibody, *red line*). (*B*) HPC-7 cells were cultured in MethoCult GF media for 7 days and significant numbers of myeloid colony-forming cell clusters were identified. Representative image of colony forming unit granulocyte macrophage colony shown. (*C*) Gene expression of S1P receptors 1–5 in cultured HPC-7 cells relative to glyceraldehyde-3-phosphate dehydrogenase by the 2^-ΔCT^ method was determined. *Bars* represent means ± SEM expression in 3 independent experiments. (*D*) Surface S1P1-receptor expression on cultured HPC-7 cells was analyzed by flow cytometry. After fixation and permeabilization of the cells, total intracellular S1P1-receptor expression also was assessed. Representative histograms of 3 independent experiments are shown (*grey area*, isotype control; *red line*, S1P1 antibody). (*E*) A total of 1 × 10^6^ HPC-7 cells were added to the top well of the Transwell and migration to varying concentrations of S1P proceeded for 4 hours. Cells migrating into the bottom well were counted using flow cytometry and expressed as a percentage of the total number of HPC-7 cells added to the top well. *Bars* represent the means ± SEM of 3 separate experiments.

**Supplementary Figure 9:**
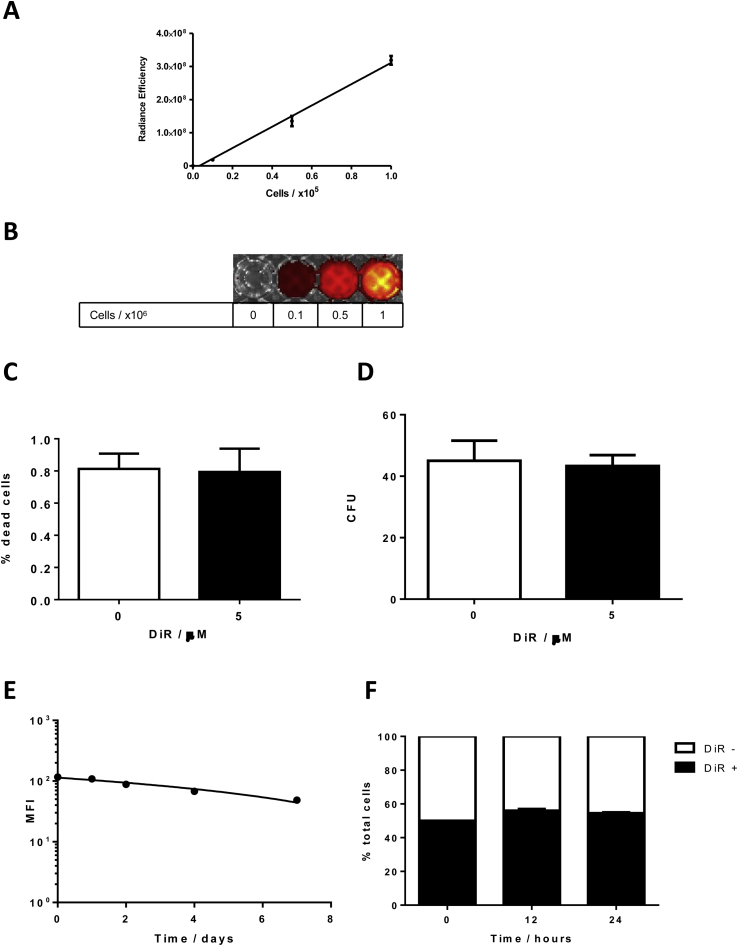
Validation of DiR staining. (*A*) Fluorescence intensity of varying numbers of cells labeled with 5 μmol/L DiR was measured using the IVIS imaging system (n = 3; means ± SD fluorescence shown). (*B*) Representative IVIS image (fluorescence intensity overlaid on photograph) of the indicated numbers of cells labeled with 5 μmol/L DiR for each. Freshly isolated KSL cells were incubated with 5 μmol/L DiR for 10 minutes at room temperature, washed, and resuspended in media. (*C*) Cell viability of labeled cells (*black bars*) was measured by viability dye exclusion determined by flow cytometry and compared with unlabeled cells (*white bars*) (n = 3; means ± SD % dead cells shown). (*D*) Colony-forming ability in labeled (*black bars*) and unlabeled (*white bars*) cells was measured by in vitro methylcellulose assay (n = 3; means ± number of colonies formed shown). (*E*) Loss of fluorescence over time: 1 × 10^6^ cultured HPC-7 cells were labeled with 5 μmol/L DiR for 10 minutes at room temperature, washed, and resuspended in complete media. A cell sample was taken at each time point and mean fluorescence intensity (MFI) was determined by flow cytometry. (*F*) Potential transfer of dye from labeled to unlabeled cells: 1 × 10^6^ cultured HPC-7 cells were labeled with 5 μmol/L DiR for 10 minutes at room temperature, washed, and resuspended in complete media together with 1 × 10^6^ unlabeled HPC-7 cells. A cell sample was taken at each time point and the percentage of fluorescent DiR-labeled cells in the sample was determined by flow cytometry (n = 3; means ± SD % fluorescently labeled cells shown). CFU, colony-forming unit.

S1P1 was the most highly expressed S1P receptor on KSL, which was found predominantly intracellularly (Supplementary Figure 6*A* and *B*). In vitro migration to S1P was dose-dependent and peaked at 1 μmol/L (Supplementary Figure 6*C*).The HPC-7 cell line had similar c-kit/sca-1 expression (with absence of lineage markers), comparable S1P/chemokine receptor expression (Supplementary Figure 7), and migrated to S1P in vitro (Supplementary Figure 8*E*). Treatment of KSL with FTY720 markedly impaired S1P-dependent cell migration (Supplementary Figure 6*D*), as did treatment with the S1P1-specific antagonist W146. After administration of FTY720 to mice with CCl_4_ injury, higher numbers of KSL were isolated from liver (1697 ± 247 vs 982 ± 110 KSL cells/liver; *P* < .05), whereas the number of circulating KSL in peripheral blood was not altered (0.36 ± 0.1 vs 0.43 ± 0.14 KSL cells/μL) (Supplementary Figure 6*E*). 1,1′-dioctadecyl-3,3,3′,3′-tetramethylindotricarbocyanine iodide (DiR) labeling of KSL did not affect their viability or proliferation and fluorescence intensity of labeled cells was maintained at 7 days without transfer of dye to unlabeled cells (Supplementary Figure 9). DiR-labeled HPC-7 cells were used to study the tissue distribution of injected HSCs, which showed rapid clearance from the lungs over the first 24–48 hours and ongoing accumulation within the liver over 48 hours followed by gradual clearance (Figure 6*A–C*). Only low levels of uptake were found in the spleen and kidneys. Administration of FTY720 did not alter initial localization of injected cells in either the liver or lungs, however, the numbers of HPC-7 remaining within the liver were 50% higher than control 4 days after injection and were 86% at 7 days (Figure 6*A–C*). Based on these findings, tissue localization of KSL cells was studied at a single time point, 4 days after injection. Greater numbers of injected KSL cells were found within the liver in mice treated with FTY720 compared with untreated controls (13,514 ± 1506 vs 7687 ± 1556 DiR-labeled KSL cells/liver; *P* < .05) (Figure 6*D*). Intravital microscopy studies showed that treatment of KSL cells with FTY720 or W146 did not increase their recruitment to the injured liver (control, 10.3 ± 0.6; FTY720, 9.6 ± 2.4; W146, 9.0 ± 1.15 cells/field of view) 60 minutes after injection (Figure 6*E* and *F*).

### Increased Retention of KSL Cells in the Injured Liver With FTY720 Enhances Their Antifibrotic Effect

**Figure 7:**
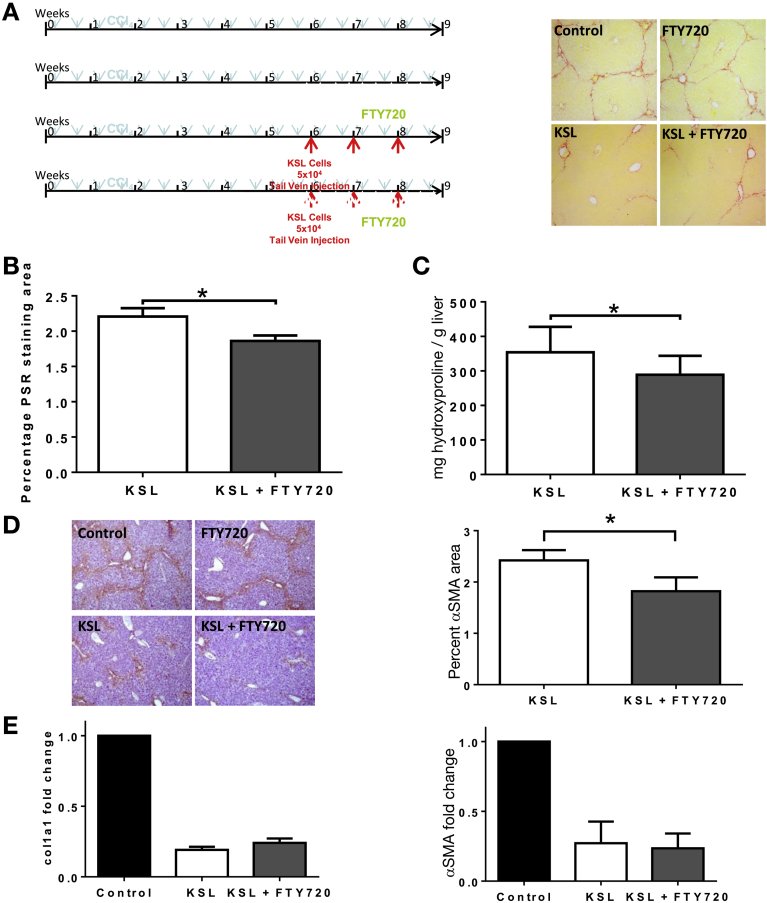
FTY720 treatment augmented the antifibrotic effect of repeated HSC injections. (*A*) Liver injury was induced in 8-week-old C57/BL6 mice by 8 weeks of twice-weekly intraperitoneal injections of CCl_4_. Mice were divided into 4 groups: injury alone, injury and FTY720, injury and injection of 5 × 10^4^ KSL cells, or injury and injection of 5 × 10^4^ KSL cells and FTY720 (n = 8–13 per group). Representative photomicrograph images of PSR staining of livers show a reduction in collagen staining of livers from KSL (± FTY720)-treated mice, compared with control mice and those receiving FTY720 only (magnification, 40×). (*B*) Quantitative analysis of picrosirius red staining showed that FTY720 treatment with KSL cell injections reduced hepatic fibrosis more than KSL injections alone (n = 8–13 per group; 5 independent experiments), with a commensurate reduction in the level of (*C*) hepatic hydroxyproline (n = 8–13 per group; 5 independent experiments). (*D*) Representative photomicrograph images of α-SMA staining (magnification, 40×) across the 4 groups indicated lower levels of α-SMA, as confirmed by morphometric analysis (n = 13 per group; 5 independent experiments). (*E*) Hepatic gene expression of α-SMA and col1a1 was suppressed in both the KSL group and the KSL and FTY720 group (n = 8–13 per group; 5 independent experiments). *P < .05.

**Supplementary Figure 10:**
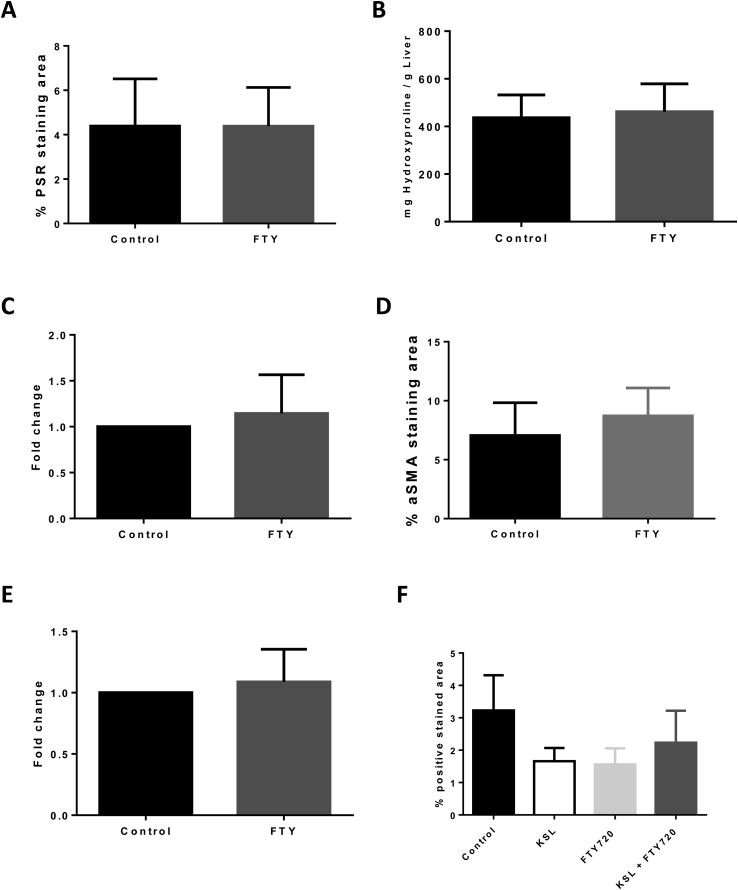
Use of FTY alone in model of chronic liver injury. Chronic liver injury was induced in age- and sex-matched C57/BL6 mice by twice weekly intraperitoneal administration of carbon tetrachloride (1 mg/kg) in mineral oil for 6 weeks, mice then were allocated randomly to a treatment group. Mice in the control group continued to receive twice-weekly injections of intraperitoneal carbon tetrachloride until week 10 and were killed 72 hours after the final injection of carbon tetrachloride. Mice in the FTY720 treatment group received twice-weekly carbon tetrachloride in the same way as control mice but in addition received 3 times weekly FTY720 (1 mg/kg) via intraperitoneal injection from the start of week 7, mice were killed at the start of week 10, 72 hours after the final injection of carbon tetrachloride. (*A*) Formalin-fixed paraffin-embedded liver tissue sections from control mice (*black bars*) and mice treated with FTY720 (*grey bars*) were stained for picrosirius red. Six random nonoverlapping images were obtained from each section and staining was quantified as a percentage of the image positive for picrosirius red using ImageJ software (n = 12–13 in each group; means ± SD % area stained shown; *P* = NS). (*B*) Hydroxyproline content in samples of liver tissue from control mice (*black bars*) and mice treated with FTY720 (*grey bars*) was determined and expressed as milligrams of hydroxyproline per gram of liver tissue (n = 12–13 in each group; means ± SD hydroxyproline content shown; *P* = NS). RNA was extracted from liver tissue of control mice (*black bars*) and mice treated with FTY720 (*grey bars*) and using quantitative reverse-transcription polymerase chain reaction gene expression of (*C*) col1a1 and (*E*) α-SMA were determined, normalized to glyceraldehyde-3-phosphate dehydrogenase expression, and relative to control mice using the 2^-ddCT^ method (n = 12–13 in each group; means ± SD fold change shown; *P* = NS). (*D*) Formalin-fixed paraffin-embedded liver tissue sections from control mice (*black bars*) and mice treated with FTY720 (*grey bars*) were stained for α-SMA. (*F*) Six random nonoverlapping images were obtained from each section and staining was quantified as a percentage of the image positive for CD45 using ImageJ software (n = 12–13 in each group; means ± SD % area stained shown; *P* = NS).

Administration of FTY720 alone did not alter hepatic fibrosis or α-SMA activation (Supplementary Figure 10). Administration of FTY720 in conjunction with repeated KSL cell injections resulted in a further 16% reduction in PSR staining (1.86% ± 0.10% vs 2.21% ± 0.11% staining; *P* < .05) (Figure 7*A* and *B*) and a 12% reduction in hepatic hydroxyproline content (289.2 ± 90.9 vs 328.9 ± 83.6; *P* = .05) (Figure 7*C*) compared with KSL cell injection alone. α-SMA staining was 21% lower (1.82 ± 0.19 vs 2.31 ± 0.22; *P* < .05) (Figure 7*D*) in the FTY + KSL group and hepatic gene expression of α-SMA and Col1a1 remained suppressed in both groups (Figure 7*E*).

## Conclusions

Despite the considerable interest in bone marrow cell therapy for liver disease there is still significant uncertainty regarding their efficacy. Moreover, there is also a lack of clarity as to which population of bone marrow cells is likely to be the most effective. In this article we show that repeated injections of a purified population of murine hematopoietic stem cells result in a marked resolution of hepatic fibrosis, in association with an increase in hepatic populations of endogenous macrophages and neutrophils. In addition, we show that abrogation of the migration of hematopoietic stem cells down an S1P gradient results in their greater hepatic retention and a further reduction in liver fibrosis, thus establishing a new therapeutic paradigm for the use of hematopoietic stem cells in this setting.

It previously has been proposed that injected bone marrow cells differentiate into antifibrotic cells of a monocyte/macrophage lineage.^11^ However, we have shown that myeloid differentiation is not required for cells to exert their antifibrotic effect in our study because injection of lymphoid progenitor cells induced a similar amount of fibrosis resolution as injections of either myeloid progenitors or HSCs. Neutrophils and macrophages, with expression of MMP9 and MMP13, are critical to the resolution of fibrosis^27^, ^28^ and significant increases in these cell populations were observed in the livers of mice receiving cell injections. The experimental design of this study, using a CD45 mismatch, confirmed that these cells were recipient-derived, representing activation of endogenous repair pathways rather than a direct antifibrotic action of the injected cells. These findings clarify that HSCs exert a paracrine effect within the injured liver, stimulating repair through recruitment of other cell populations, and indeed HSCs have been shown to be potent secretory cells, mediating effector properties through cytokine stimulation of immune cell populations.^29^ Further study of the chemokines and cytokines (eg, CCL2, interleukin 10, tumor necrosis factor-like weak inducer of apoptosis [TWEAK]) secreted by HSCs may further define the mechanisms involved. Notably, our data indicate that murine monocytes, which may mediate the antifibrotic effect of HSCs, are recruited to CCL2 and also to murine HPC-7 cells in a CCR2-dependent manner, suggesting that a potential mechanism by which monocytes are recruited/positioned within the injured liver relates to hematopoietic stem cell–expressed/secreted chemokine ligands. There were no differences in the proportion of macrophage subsets within the liver, or as a ratio of Ly-6C^hi^/Ly-6C^lo^ (M1-like to M2-like) macrophages, after KSL cell infusion. Similarly, no differences were seen in peripheral blood analysis. As recognized by the literature, surface marker expression of macrophages is likely to be more complex and dynamic and thus even extensive panels do not completely characterize the full phenotype of macrophages in vivo.^30^ The integral role of macrophages in mediating both the generation and resolution of fibrosis has been shown by other groups, including our previous work.^31^, ^32^

It is assumed that homing of HSCs to the injured liver is required for them to exert their antifibrotic actions, although this has not been proven. Increased recruitment of hematopoietic stem cells to the liver has been reported in response to stress-induced signals, such as increased expression of stromal-derived factor-1, MMP9, and hepatocyte growth factor, which recruit human CD34+ progenitors.^33^ We previously reported that the adhesion of human hematopoietic (CD34+) stem cells to human liver compartments is integrin- and CD44-dependent and modulated by CXC chemokine receptor 3 and CXC chemokine receptor 4.^34^ Our BM transplantation studies confirmed that liver injury with CCl_4_ increases the number of BM-derived HSCs in the liver, but without any change in the number of HSCs in other organs. There have been a variety of different approaches taken to the administration of stem cell therapy in both human beings and animal models, the peripheral venous route represents the safest and most feasible mode of delivery and we have shown this to be effective because cells injected into a peripheral vein exert a beneficial therapeutic effect. Analysis of the distribution of injected HSCs showed their accumulation within the lungs immediately after injection followed by a rapid clearance, whereas recruitment to the liver increased over the first 48 hours with a more gradual reduction in cell numbers subsequently. Seven days after injection the number of injected HSCs remaining within the liver was very small and explains the absence of detectable numbers of cells on tissue section analysis. The whole-organ analysis in this study provided an accurate assessment of cell numbers because previous studies have used tissue section analysis, which may not quantify small numbers of cells accurately or provide appropriate comparisons between organs, for example, overestimating the number of injected cells within the lungs.

S1P has been recognized to mediate recirculation of HSCs down S1P gradients from tissue back into lymph and the circulation,^20^ and we show that S1P levels and activity are increased in the injured liver, thus partially reversing the normal S1P liver tissue:lymph gradient. Our data suggest that the increase in hepatic S1P levels is caused by up-regulation of SphK1 in liver sinusoidal endothelial cells with less significant changes in other enzymes involved in S1P processing. To establish whether S1P was acting to increase recruitment of bone marrow–derived cells to the liver, we performed intravital microscopy (Figure 6*E* and *F*) with blockade of the S1P axis using either a function-blocking antibody to S1P1 receptor or FTY720, neither of which altered the number of exogenously administered bone marrow–derived cells seen within the liver over a 60-minute period. We therefore hypothesize that the increased hepatic S1P levels act primarily to retain HSCs within the liver by preventing their egress down the existing S1P liver–lymph gradient rather than priming the liver to recruit bone marrow–derived cells, which is in keeping with the observations of others in normal physiological conditions.^20^, ^35^

To exploit this modulation of the S1P pathway we used pharmacologic manipulation with the partial S1P_1_-receptor agonist, FTY720. FTY720 impaired the migration of HSCs in vitro and when administered to mice with liver injury resulted in accumulation of HSCs within the liver. Although FTY720 did not affect trafficking of HSCs within the first 48 hours after injection, increased numbers of injected HSCs were present in mouse livers at later time points compared with placebo controls. This supports our observations that HSC recruitment to the injured liver is not mediated through S1P receptors and that the increased number of HSCs in the liver is a result of increased retention of HSCs within the liver, rendered unresponsive to the S1P gradient by FTY720. The use of isolated KSL cells in these tracking studies was limited by the difficulties isolating the large number of cells required, and thus the findings from experiments using the HPC-7 cell line were used to design experiments with isolated KSL cells at a single time point. This study, with KSL cells, also confirmed that mice receiving FTY720 had a greater number of cells within the liver 4 days after infusion.

Although administration of FTY theoretically could reduce homing to any organ, our tracking data (Figure 6*B* and *C*) indicated no change in the number of KSL cells found in the lungs. We therefore believe that the reason FTY720 increases the number of KSL cells in the liver, without an effect on other organs, is the result of a combination of a reduced liver–lymphatic S1P gradient (caused by the induction of liver injury, which increases hepatic S1P levels) and a reduced tendency to migrate down an S1P axis owing to the pharmacologic action of FTY720. Notably, FTY720 has a range of systemic actions that in their own right may be antifibrotic, such as inhibiting cytosolic phospholipase A2 and antagonizing cannabinoid receptor 1,^36^ although in this study it had no antifibrotic effect when used in isolation. The antifibrotic effect of injected HSCs was augmented by approximately 20% with the addition of FTY720, and although the clinical significance merits further study, any optimization of the effect of this very rare population of cells should be considered advantageous. The optimal number of cells required to achieve an antifibrotic effect remains uncertain; in our studies we chose to administer lower numbers of cells than in previous studies and it is not clear whether the number of cells administered was proportional to the antifibrotic effect or whether there is a plateau effect beyond which no greater effect would be observed.

Our data show the potent antifibrotic actions of purified murine hematopoietic stem cells, and also indicate that enhanced exposure of the target organ, the injured liver, to infused HSCs is associated with an augmentation of their antifibrotic effect. These findings support the development of clinical trials of stem cell therapy in human beings.

## Figures and Tables

**Supplementary Table 1:** Table of Antibodies Used

Antibodies	Clone	Conjugate	Source
Live/dead		APC-CY7 e780	Life Technology
CD45.2	104	eFluor 506	Ebio
Foxp3	FJK-16s	APC	Ebio
CD3	17A2	eFluor 450	Ebio
CD4	GK1.5	FITC	Ebio
NkP46	29A1.4	PE	Ebio
CD8	53-6.7	BV 786	Biolegend
LY6C	HK1.4	PE	Ebio
LY-6G/Gr-1	1A8-Ly6g	APC	Ebio
CD3	17A2	BV650	Biolegend
B220	Ra3-6B2	eFluor 450	Ebio
CD11b	M1/70	PE-e610	Ebio
F4/80	BM8	PE-CY7	Ebio
CD45.2	104	FITC	Ebio
CD16	93	Purified	Ebio

FITC, fluorescein isothiocyanate.

## Supplementary Materials and Methods

### Tissue Preparation

Blood samples were obtained by cardiac puncture under general anesthesia and mice were killed by cervical dislocation. Bone marrow cells were flushed from the medullary cavities of the femur and tibia with Dulbecco's modified Eagle medium + 10% fetal calf serum using a 25G needle. A single-cell suspension was prepared by passing the cells through a 40-um filter (BD Falcon, London, UK) and analyzed by flow cytometry or used for cell sorting. Mouse tissues were harvested and perfused with phosphate-buffered saline, dissociated, and digested with 2 mg/mL collagenase IV (Sigma). Mononuclear cells were isolated by density centrifugation using an Optiprep (Sigma) gradient and the resulting cell suspension was used for flow cytometry. Samples of frozen human liver tissue were obtained from patients at the University Hospital Birmingham with chronic liver disease undergoing liver transplantation or from normal organ donor liver not used for transplantation with written informed consent and local ethics committee approval.

### Immunohistochemistry

Liver specimens were fixed in 4% paraformaldehyde, embedded in paraffin, and cut into 4-μm sections. Slides were deparaffinized and antigen retrieval was performed using a citrate buffer pH 6.0. Primary antibody was added for 1 hour: anti–α-SMA (1:400; Abcam), anti-F4/80 (1:200; AbD Serotec), anti-Ly6G (1:500; AbD Serotec), anti-CD45.2 (1:200; eBioscience) followed by secondary antibody (ImmPRESSS peroxidase anti-rabbit IgG or anti-rat [mouse adsorbed] IgG; Vector Labs), and expression was visualized using ImmPACT DAB reagent (Vector Labs). Sections were counterstained with hematoxylin and mounted using DPX (Fisher Scientific).

### Hydroxyproline Assay

Frozen liver tissue samples were weighed and protein was precipitated by the addition of 1:1 trichloroacetic acid:water and incubated on ice for 30 minutes. Samples were hydrolyzed with 6 N HCl in sealed borosilicate tubes and incubated at 120°C for 16 hours and at 80°C until completely desiccated. The precipitate was resuspended in water and filtered. Chloramine T solution was added at room temperature for 20 minutes, followed by the addition of Ehrlich’s solution and incubation at 65°C in a water bath for 15 minutes. After cooling to room temperature, fluorescence readings at 561 nm were taken from samples and standards, and hydroxyproline content was expressed as mcg per gram of liver tissue.

### Flow Cytometry and Cell Sorting

Erythrocytes were lysed using ACK red cell lysis buffer (Sigma). After incubation with anti-CD16/32 antibody (1:20; eBioscience) to eliminate nonspecific fragment crystallizable receptor binding, cell suspensions were labeled with the relevant primary antibodies (all 1:100): anti-CD11b–fluorescein isothiocyanate (M1/70), anti-CD5–fluorescein isothiocyanate (53-7.3), anti-CD8a–fluorescein isothiocyanate (53-6.7), anti-Ter119–fluorescein isothiocyanate (TER119), anti-CD45R–fluorescein isothiocyanate (RA3-6B2), anti-Gr1–fluorescein isothiocyanate (RB6-8C5), anti-CD117 (c-kit)–PE, anti-CD117 (c-kit)-Pacific Blue, anti-CD117 (c-kit)-APC-Cy7 (all 2B8), anti-Sca1–APC (D7), anti-CD127 (interleukin 7Ra)-PE (A7R34), anti-EDG1 (S1P1)-PE (T4-H28), anti-CD45.1-PE (A20), anti-CD45.2-APC (104) and incubated on ice for 30 minutes. Nonviable cells were excluded using LIVE/DEAD stain (Invitrogen). Absolute cell counts were determined using Trucount beads (Invitrogen) and data were analyzed using a CyAn adenosine diphosphate flow cytometer with Summit (London, UK) 4.3 software. Cell sorting was performed using a MoFloCell Sorter (Dako, London, UK) with Summit 5.2 software. Cells to be injected were sorted directly into sterile phosphate-buffered saline and used immediately, whereas cells for polymerase chain reaction analysis were sorted directly into RNeasy Lysis buffer ready for RNA extraction.

### Cell Isolation From Blood and Liver and Flow Cytometric Analysis

Blood was collected through cardiac puncture into EDTA tubes. Red blood cells were lysed with 5 minutes of lysis buffer. Isolated cells then were washed twice and resuspended in magnetic-activated cell sorting (MACS) buffer (2% fetal calf serum and 1 mmol/L EDTA). Liver homogenate was filtered after being homogenized with a 5-mL syringe plunger. Single cells then were overlaid on density gradient of Optiprep and centrifuged at 1000*g* for 25 minutes at room temperature. After 2 washes, isolated cells then were suspended in MACS buffer.

Antibodies used for flow cytometry are listed in Supplementary Table 1. Single-cell suspensions were blocked with anti-mouse fragment crystallizable region (FcR) antibody (CD16/CD32) for 15 minutes at 4°C in MACS buffer, and stained with primary conjugated antibodies. For internal staining, cells were fixed and permeabilized with a Foxp3/permeabilization kit (eBioscience). Flow cytometry was performed with Fortessa (BD Biosciences) with forward- and side-scatter gates set to exclude nonviable cells. Data were analyzed with Flowjo software (version 10.1).

Flow cytometric analysis showed liver macrophages (gated CD45^+^CD3^-^CD11b^+^F4/80^+^Ly-6G^-^), resident (res)macrophages (gated CD45^+^CD3^-^CD11b^int^F4/80^+^), M1-macrophages (gated CD45^+^CD3^-^CD11b^+^F4/80^+^Ly-6G^-^Ly6C^hi^), and M2-macrophages (gated CD45^+^CD3^-^CD11b^+^F4/80^+^Ly-6G^-^Ly6-C^lo^).

### Quantitative Reverse-Transcription Polymerase Chain Reaction

RNA extraction was performed using the RNeasy Mini (liver tissue) and Micro (isolated cells) kits (Qiagen) according to the manufacturer’s instruction, concentration and purity were checked using a Nanodrop 1000 (Thermo Scientific). Complementary DNA was transcribed from RNA using iScript reverse-transcriptase (Bio-Rad). Reverse-transcription polymerase chain reaction was performed on an ABI Prism 7900HT or Stratagene MX3000p using TaqMan assay mix in predesigned TaqMan assays as detailed in the Supplementary information (Applied Biosystems). The program consisted of activation at 95°C for 10 minutes, followed by 40 cycles of denaturing (95°C for 10 seconds) and annealing (60°C for 60 seconds) with fluorescence detection at the end of each cycle. Normalization to glyceraldehyde-3-phosphate dehydrogenase using the 2^-ΔΔCT^ method was performed and expression relative to the appropriate control was calculated.

### Serum Albumin

Serum albumin concentrations were determined using the Quantichrom BCG Albumin Assay (Bioassay Systems) according to the manufacturer’s instructions.

### Western Blot

Whole-liver protein extracts were quantified using Bradford reagent and 50 μg used for Western blot. The primary antibody was either anti-SphK1 (1:500; Abcam) or anti–glyceraldehyde-3-phosphate dehydrogenase (1:2000; Sigma) incubated overnight and horseradish-peroxidase–conjugated goat anti-rabbit IgG antibody (1:10,000; Sigma) secondary antibody incubated for 30 minutes. Pierce chemiluminescent peroxidase substrate and Hyperfilm ECL were used to visualize protein expression.

### Transwell Migration

Male C57/BL6 mice were culled by cervical dislocation and their tibias and femurs were dissected from the cadavers for monocyte isolation. Ends of bones were trimmed and the bone marrow was flushed out with MACS separation (MACs) buffer (phosphate-buffered saline + 2 mmol/L EDTA + 0.5% bovine serum albumin). After centrifugation and cell count, monocytes were selected from the cell suspension using magnetic labeling and separation. After washing, the cells were resuspended in MACs buffer and incubated for 5 minutes at 4°C with FcR Blocking Reagent and Monocyte Biotin-Antibody cocktail. The cells then were washed before being incubated with Anti-Biotin Microbeads for 10 minutes at 4°C and then passed through an LS column. The unlabeled cells represented the monocyte population and passed through the column. Monocytes then were incubated with cell tracker green for 20 minutes at room temperature (the cell tracker green was diluted 1:1000 to provide a concentration of 5 μmol/L). CCL2 was prepared in migration media (Roswell Park Memorial Institute + 1% PSG + 0.1% bovine serum albumin) to a concentration of 100 ng/mL, and 150 μL of this added to the receiver plate along with media alone, unstained monocytes, and HPC-7 cells. Monocytes were washed and resuspended in migration media to generate a density of 2 × 10^5^ cells/50 μL. A total of 50 μL of stained monocytes then were added to the Transwell insert and the plate was incubated for 4 hours at 37°C. After this incubation, samples were collected from the receiver plate and placed into separate fluorescence-activated cell sorter tubes. Cells then were washed, resuspended in MACs buffer, and quantified flow cytometrically.

### Near-Infrared Fluorescence Imaging

DiR (Invitrogen) was dissolved in methanol (1 mg/mL) and stored in the dark. A total of 5 μL/mL of DiR stock solution was added to cells at 1 × 10^6^/mL in complete StemPro media (London, UK) and incubated in the dark for 30 minutes at 37° C. Cells were washed and resuspended in 100–200 μL sterile phosphate-buffered saline for re-injection.

Near-infrared imaging was performed using the IVIS Spectrum Imaging System (autoexposure; medium binning; excitation wavelength, 745 nm; emission wavelength, 800 nm). Fluorescence of individual organs was determined by Living Image Software (Perkin Elmer) using region-of-interest analysis after subtraction of background and expressed as radiance efficiency (calculated as follows: radiance of subject/illumination intensity) to eliminate variability in the excitation light across the field of view.

### Intravital Microscopy

C57BL6 mice with 8 weeks of CCl_4_ liver injury underwent ketamine/xylazine anesthesia and carotid artery cannulation. Laparotomy was performed and the liver was exteriorized and placed on the stage of an Olympus IX81 inverted microscope. One 10× power field of view was selected for imaging. HPC-7 cells were labeled with 5 μmol/L CFSE and injected, 1-minute video recordings of the selected field of view were taken every 5 minutes until 60 minutes and analyzed for adhesion (stationary >30 seconds) of labeled cells using Slidebook software (Intelligent Imaging Innovations, London, UK).
